# Intranasal vaccination with *Listeria ivanovii* as vector of *Mycobacterium tuberculosis* antigens promotes specific lung-localized cellular and humoral immune responses

**DOI:** 10.1038/s41598-019-57245-6

**Published:** 2020-01-15

**Authors:** Ming-juan Jiang, Si-jing Liu, Lin Su, Xiang Zhang, Yong-yu Li, Tian Tang, Chuan Wang

**Affiliations:** 1 West China School of Public Health and Healthy Food Evaluation Research Center, Sichuan University, Chengdu, P. R. China; 2 Food Safety Monitoring and Risk Assessment Key Laboratory of Sichuan Province, Department of Public Health Laboratory Sciences, West China School of Public Health, Sichuan University, Chengdu, P. R. China

**Keywords:** Mucosal immunology, Vaccines

## Abstract

We have previously demonstrated that a recombinant *Listeria ivanovii* (LI) strain expressing the ESAT-6 or Ag85C protein of *Mycobacterium tuberculosis* (Mtb) as a tuberculosis (TB) vaccine candidates induced antigen-specific cellular immune responses after intravenous immunization of mice. However, whether such recombinant strains could induce desired immune responses in the lung, where TB infection occurs, is not clear. In this paper, C57BL/6 J mice were intranasally vaccinated with attenuated LI*ΔactAplcB*-*Rv3875* (*Δ* refers to gene deletion in the bacterial genome) or LI*ΔactAplcB*-*Rv0129c*, the two vaccine candidates that utilize LI as an antigen delivery vector. Bacterial load in the target organs, histological changes in the infected organs, the percentage of specific cytokine-secreting T cells in the lung and spleen, IgG levels in the serum and secretory IgA (SIgA) levles in bronchoalveolar lavage (BAL) fluid were determined at specific days post inoculation (dpi). The results showed that both strains were mainly confined to the lung and were eliminated at 10 dpi. The histological damage caused by the infection in the lung was slight and recovered by day 5. Intranasal vaccination of the mice twice at an interval of 4 weeks notably elicited TB antigen-specific CD4^+^ and CD8^+^ T cell responses in the lung and SIgA secretion in the pulmonary mucosa, and significantly enhanced the percentage of double-functional CD8^+^ T cells (IFN-γ^+^ TNF-α^+^ CD8^+^). To our knowledge, this is the first report regarding the used of LI vector vaccines to induce promising lung-localized cellular and humoral immune responses by intranasal vaccination. These data suggest that LI could be a novel and promising live vector to construct an intranasal vaccine against respiratory diseases.

## Introduction

Tuberculosis (TB) is a leading contagious disease causing significant global morbidity and mortality. Calmette-Guerin (BCG) is the only licensed vaccine for preventing childhood tuberculous meningitis and military tuberculosis worldwide^1^. However, its efficacy highly varies in adults, ranging from 0% to 80% in different regions^2^. Given that BCG is intradermally delivered, it mainly induces systemic immune responses that depend on T helper type1 (Th1) CD4 + T cell responses^3,4^. Because TB primarily establishes in the lung, vaccines that are capable of inducing respiratory mucosal immunity and pulmonary immune responses will confer better protection against pulmonary TB^5,6^

*Listeria monocytogenes* (LM) is capable of multiplying in macrophages and thus elicits robust CD4^+^ and CD8^+^T cell-mediated immune responses^7–9^. Over the few past decades, LM has been extensively utilized as a powerful vaccine vector for T cell-mediated immunity, especially for cancer immunotherapy^10–12^. Additionally, LM is a potent inducer of antigen-specific cytotoxic lymphocytes (CTLs) that directly attack intracellular pathogens such as *Mycobacterium tuberculosis* (Mtb)^13^. However, LM is pathogenic to humans and causes serious listeriosis in susceptible humans especially the immunocompromised people^9^.

*Listeria ivanovii* (LI), a species in the genus *Listeria*, shares a similar intracellular life cycle and virulence determinants as LM^14,15^, but it possesses notably decreased virulence and induces infections that only in ruminants^16^. Hence, LI is a safer live vaccine vehicle compared to that of LM. Recently, two recombinant attenuated LI strains expressing the ESAT-6 or Ag85C protein of Mtb were constructed, and an antigen-specific CD8^+^ T cell-mediated immune response was obtained via intravenous vaccination^17^. However, whether such recombinant strains could induce lung-localized and systemic cellular and humoral immune responses when intranasally administered to mice is still unknown.

Our previous studies showed that LI infection in mice is restricted to the lung after intranasal administration^18^, which leads us to hypothesize that LI might be a potential vaccine vector for developing vaccines against pulmonary infections. Investigating the ability of recombinant LI strains integrated with heterologous antigens to induce immune responses via intranasal vaccination is valuable in evaluating LI as a good antigen delivery vehicle for vaccines against respiratory tract infections. In this study, we evaluated the biosafety and immunogenicity of intranasal vaccination using two recombinant LI strains, LI*ΔactAplcB-Rv3875* (LI*Δ-Rv3875*) and LI*ΔactAplcB-Rv0129c* (LI*Δ-Rv0129c*)^17^, to verify our hypothesis. We found that both strains induced lung-localized immune responses, including antigen-specific cellular immune responses and the secretion of secretory IgA (SIgA). To our knowledge, this is the first report regarding the use of LI vector vaccines to induce promising lung-localized cellular and humoral immune responses by intranasal vaccination. Our research suggests that LI might be a novel and optimal live vector for respiratory mucosa vaccines.

## Results

### Analysis of protein expression

**Figure 1:**
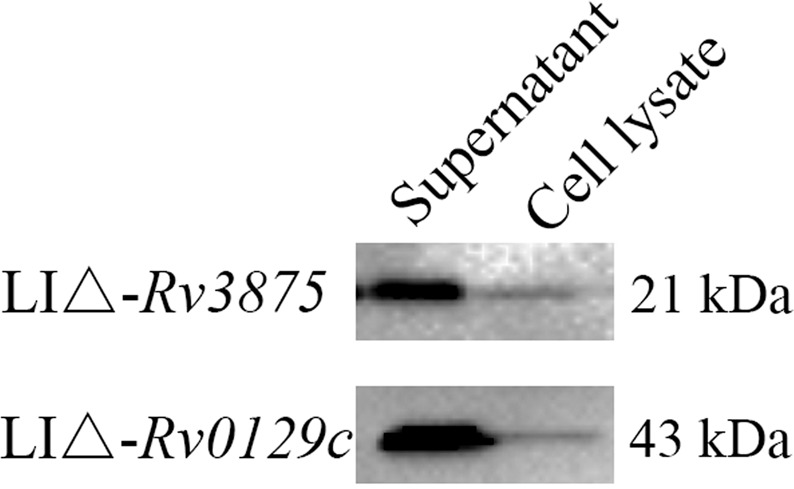
Western blotting of culture supernatant and cell lysate from LIΔ*-Rv3875* and LIΔ*-Rv0129c* using the anti-ESAT6 or anti-*Mycobacterium tuberculosis* Ag85 antibodies.

Western blotting was performed to test the protein expression of expected antigens (ESAT-6 or Ag85C) in the two recombinant LI strains. Figure 1 showed the positive results.

### The bacterial load in organs after vaccination with *LIΔ-Rv3875* or *LIΔ-Rv0129c*

**Figure 2:**
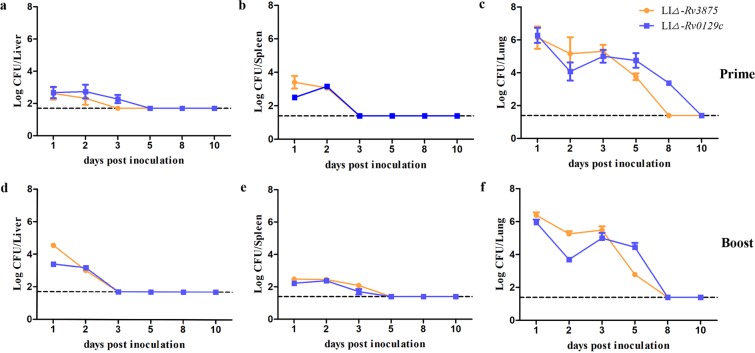
Bacterial loads in the liver, spleen and lung after prime (**a–c**) and boost (**d–f**) intranasal administration. Mice were intranasally inoculated with 10^8^ CFU LI*Δ-Rv3875*(●) or 10^8^ CFU LI*Δ-Rv0129c* (█). The bacterial loads in the liver (**a,d**), spleen (**b,e**) and lung (**c,f**) were determined on the indicated days. There was no significant difference between the two strains. The dotted lines represent the detection limits in each experiment. The experiments were performed with biological triplicates. Each point represents the mean ± SEM for a group of six mice from one independent experiment.

C57BL/6 J mice were inoculated with 10^8^ CFU of LI*Δ-Rv3875* or LI*Δ-Rv0129c*, and the bacterial load in the liver, spleen and lung was detected at different days post inoculation (dpi). After prime immunization, the bacterial loads of both strains in the liver (Fig. 2a) and spleen (Fig. 2b) reached a peak within 24~48 h post inoculation and then quickly declined to undetectable levels at 3 dpi. The highest load of both strains in the liver and spleen seemed low with no more than 10^4^ CFU. However, in the lung (Fig. 2c), both strains showed the highest load at 10^6^ CFU at 1 dpi, maintained a relatively high load (approximately 10^4^ to 10^5^ CFU) for 5 days, and finally decreased to below the detectable limit at 8~10 dpi.

After secondary inoculation, the bacterial loads of both strains in the liver reached a peak at 1 dpi and then declined (Fig. 2d). In the spleen, both strains showed a slightly longer proliferative process than that of prime immunization. The loads of the two strains declined to undetectable levels at 5 dpi. The bacterial growth curves in the lung were similar between prime- and boost-immunized mice. Both strains persisted in the lung for 5 days and dropped to below the detection limit at 8 dpi. Taken together, the bacterial loads in the liver and spleen were dramatically lower than those in the lung. Both strains mainly colonized at the lung but were eliminated at 10 dpi after intranasal immunization.

### Histopathological analysis of infected organs after vaccination with *LIΔ-Rv3875* or *LIΔ-Rv0129c*

**Figure 3:**
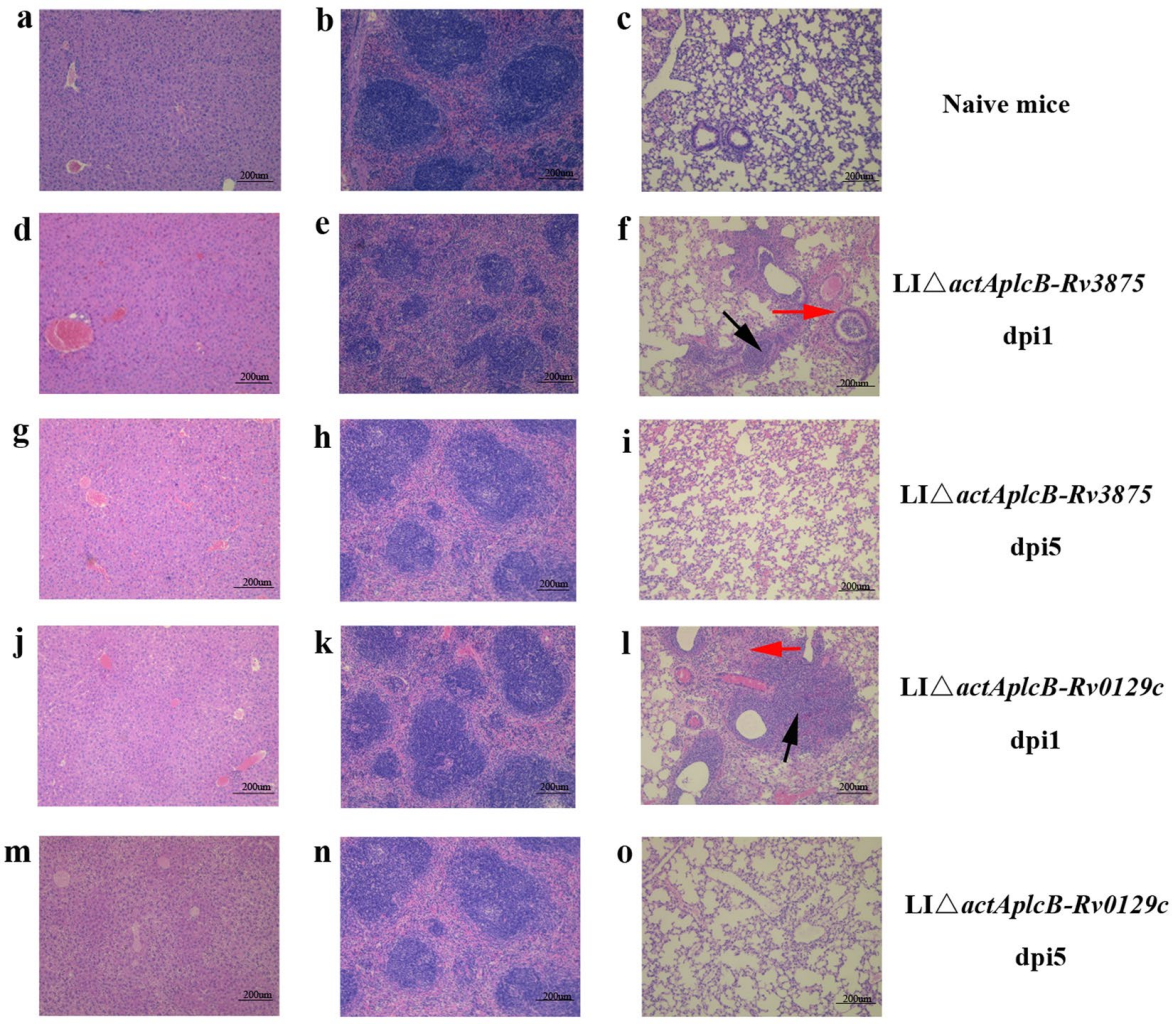
Histopathological analysis of the liver, spleen and lung after prime intranasal administration. Histological sections of the liver, spleen and lung at 1 dpi or 5 dpi were prepared and stained with haematoxylin-eosin. Histopathological changes in the liver, spleen and lung were observed and imaged under a 100 × microscope. Representative pathology is indicated by arrows. The experiments were performed with three biological replicates. The images presented are representatives of changes observed in the mice.

**Figure 4:**
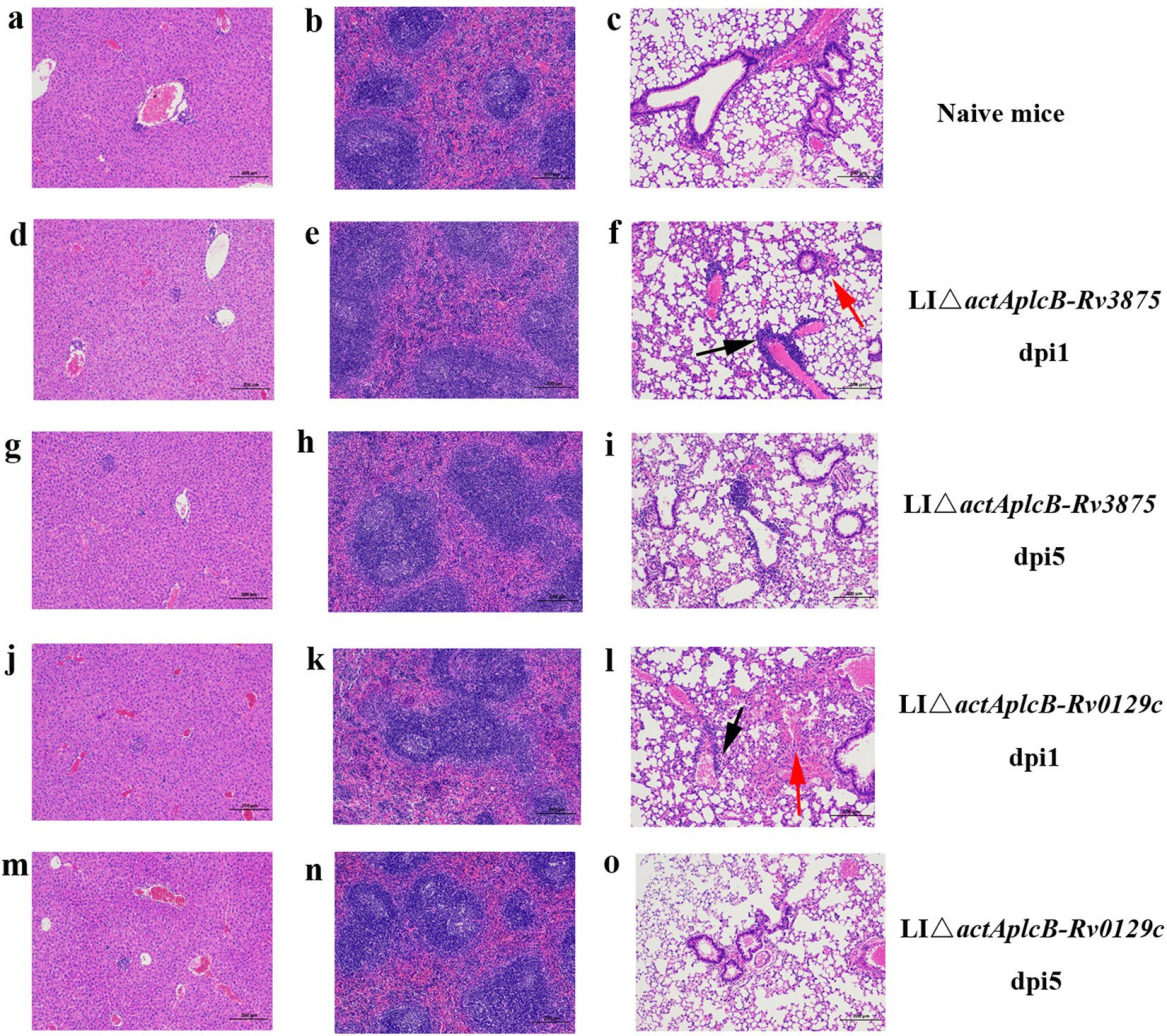
Histopathological analysis of the liver, spleen and lung after boost immunization. Histological sections of the liver, spleen and lung at 1 dpi or 5 dpi were prepared and stained with haematoxylin-eosin. Histopathological changes in the liver, spleen and lung were observed and imaged under a 100 × microscope. Representative pathology is indicated by arrows. The experiment was repeated three times. The images presented are representatives of changes observed in the mice.

As shown in Fig. 3f,l, at 1 dpi, the main histopathological changes in the lung after prime immunization were inflammatory cell infiltration (black arrow indicated) and haemorrhage (red arrow), and the infection was resolved by 5 dpi (Fig. 3i,o). The same phenomenon was observed in the lung after boost immunization (Fig. 4f,i,l,o). No obvious histological changes were observed in the liver and spleen after prime or boost immunization. Histopathological results were consistent with the bacterial growth curves in the target organs, confirming that the two strains were mainly localized, mildly affected the lung and barely infected the spleen and liver following intranasal inoculation.

### Antigen-specific cytokine production after prime immunization

**Figure 5:**
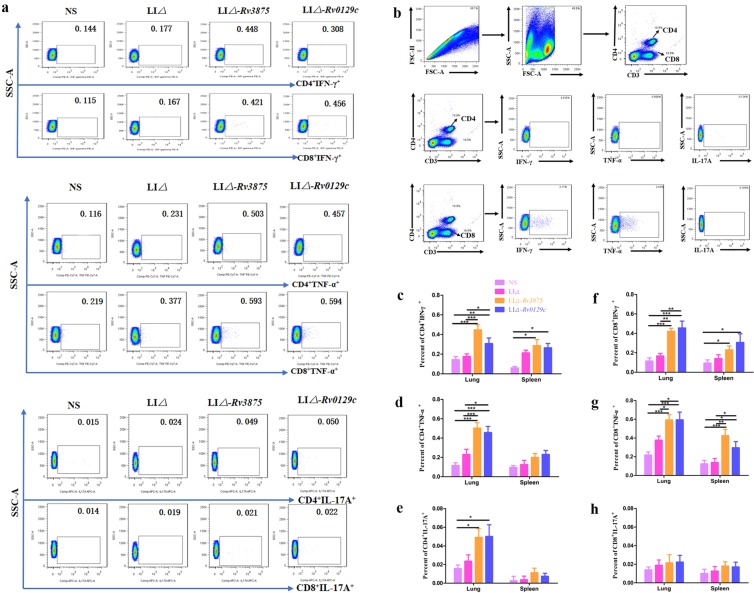
Comparison of antigen-specific cytokine production in the lung and spleen after primary vaccination with recombinant strains. Mice were intranasally administered 10^8^ CFU LI*Δ-Rv3875*, LI*Δ-Rv0129c*, or LI*Δ* or NS. Two weeks after vaccination, the spleen and lung were harvested. (**a**) Representative dot plots of IFN-γ-, TNF-α- or IL-17A-positive CD4 and CD8 T cells in the lung that were stimulated by mixed peptides after prime vaccination. The numbers in each dot plot indicate the percentages of corresponding positive cells in the CD4+ or CD8+ T cell population. (**b**) The gating strategy for analysing the cytokine-positive CD4+ or CD8+ T cells by flow cytometry is shown. The gating strategy was the same for lung and spleen samples. (**c–h**) The percentage of IFN-γ-, TNF-α- or IL-17A-positive CD4^+^ (**c**–**e**) or CD8^+^ T cells (**f–h**) was determined by flow cytometry. *p < 0.05, **p < 0.01, ***p < 0.001. The experiment was repeated three times. The dot plots presented are representatives of flow cytometry data. Each bar represents the mean ± SEM per group of seven mice from one independent experiment.

C57BL/6 J mice were intranasally immunized with LI*Δ-Rv3875* or LI*Δ-Rv0129c*, and control groups (LI*ΔactAplcB-lacZ* (LI*Δ*) or normal saline (NS)) were included. At 14 dpi, mouse lung cells and splenocyte suspensions were prepared for intracellular cytokine staining. As shown in Fig. 5, LI*Δ-Rv3875* and LI*Δ-Rv0129c* induced significantly higher levels of IFN-γ and TNF-α in CD4^+^ and CD8^+^ T cells in the lungs compared with those of both controls, while the IL-17A response was weak, as there were no significant differences between the groups. In the spleen, the T cell response was relatively low, although a certain enhanced level of antigen-specific TNF-α was detected in CD8^+^ T cells in the LI*Δ-Rv3875* and LI*Δ-Rv0129c* groups compared with those of the controls. These results indicated that LI*Δ-Rv3875* and LI*Δ-Rv0129c* mainly elicited localized lung local immune responses after prime intranasal administration.

### Boost immunization in the lung

**Figure 6:**
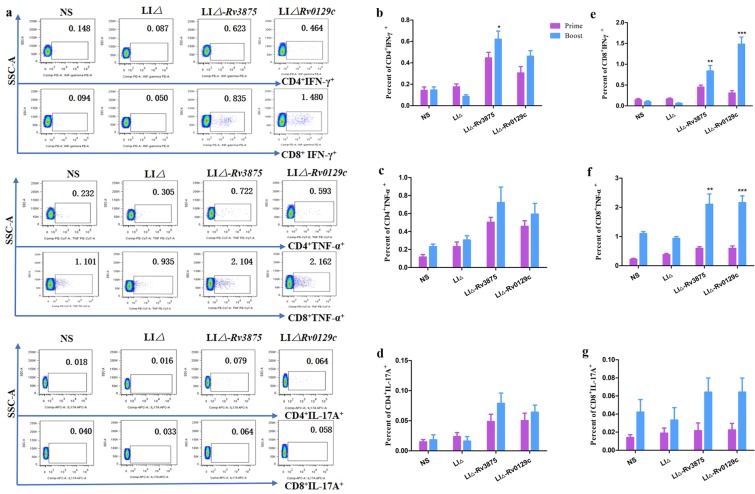
Enhanced antigen-specific cytokines in the lung after boost vaccination. Four weeks after primary vaccination, the mice were intranasally administered the same dose of the same strains. (**a**) Representative dot plots of IFN-γ-, TNF-α- or IL-17A-positive CD4 and CD8 T cells in the lung that were stimulated by mixed peptides after boost vaccination. The numbers in each dot plot indicate the percentages of corresponding positive cells in the CD4 + or CD8 + T population. (**b–g**) The percentage of IFN-γ-, TNF-α- or IL-17A-positive CD4^+^ (b-d) or CD8^+^ T cells (**e–g**) was determined by flow cytometry. *p < 0.05 (boost *vs*. prime), **p < 0.01 (boost *vs*. prime), ***p < 0.001 (boost *vs*. prime). The experiment was repeated three times. The dot plots presented are representatives of flow cytometry. Each bar represents the means ± SEM per group of seven mice from one independent experiment. The gating strategy for analysing cytokine-positive CD4 + or CD8 + T cells by flow cytometry is the same as described in Fig. 5b.

To further assess that whether the two strains could induce increased cellular immune responses after boost vaccination, we vaccinated the mice 4 weeks after primary vaccination. As shown in Fig. 6, antigen-specific cytokine responses after boost immunization were much stronger than those of prime immunization, especially in CD8^+^ T cells. The percentages of CD8^+^IFN-γ^+^ cells in the LI*Δ-Rv3875* and LI*Δ-Rv0129c* immunization groups were two or three times of those of prime administration. The percentages of CD8^+^ TNF-α^+^ cells in the two groups after boost administration were four times those of prime administration.

### Polyfunctional T cell responses in the lung after boost immunization

**Figure 7:**
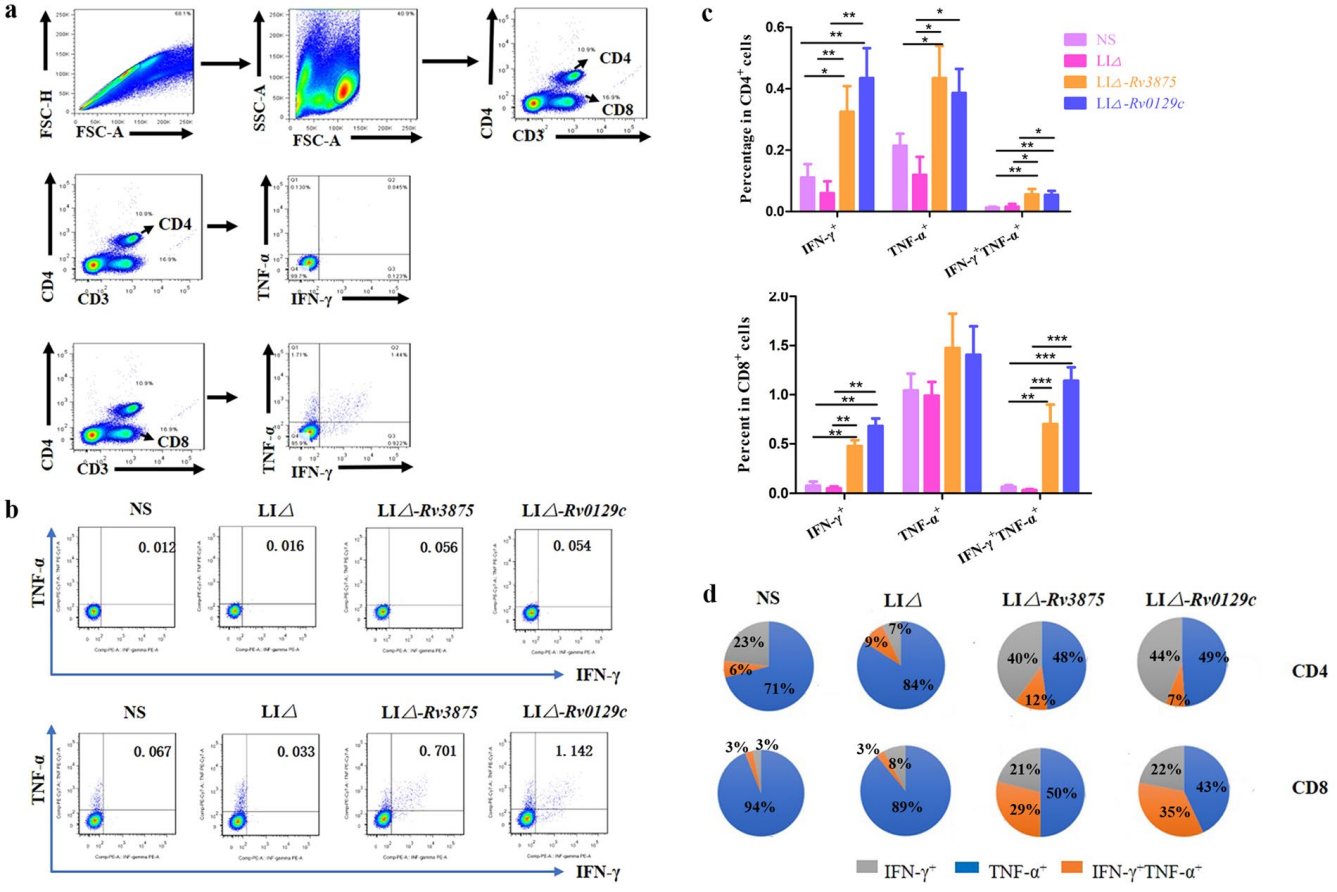
High levels of IFN-γ and TNF-α double-positive T cell induction in the lung after boost administration. Two weeks after boost immunization, lung cells were prepared and flow cytometry was used to analyse the frequencies of CD4^+^ or CD8^+^ T cells that produced one or two cytokines. (**a**) The gating strategy for analysing double-positive CD4 + or CD8 + T cells by flow cytometry is shown. (**b**) Representative dot plots of IFN-γ and TNF-α double-positive T cells are shown. (**c**) Percent of CD4^+^ or CD8^+^ T cells that produce one or two cytokines are shown in the bar graph. (**d**) The proportions of the three types of CD4^+^ or CD8^+^ T cells are shown in the pie chart. *p < 0.05, **p < 0.01, ***p < 0.001. The experiment was repeated three times. The dot plots presented are representatives of flow cytometry data. Each bar or pie section represents the mean ± SEM per group of seven mice from one independent experiment.

Polyfunctional T lymphocytes are important for preventing TB, and so we further analysed the induction of IFN-γ and TNF-α double-positive T cells in the lung. Figure 7 shows that the percent of IFN-γ single positive CD4^+^ or CD8^+^ T cells in the LI*Δ-Rv3875* and LI*Δ-Rv0129c* immunization groups were significantly higher than those in the control groups. Additionally, mice vaccinated with LI*Δ-Rv3875* or LI*Δ-Rv0129c* produced significantly more antigen-specific IFN-γ^+^ TNF-α^+^ double-positive CD4^+^ or CD8^+^ T cells than the control groups. Both vaccine groups showed a notably higher proportion of IFN-γ^+^TNF-α^+^ double-positive CD8^+^ T cells compared to those of the control groups, indicating that the IFN-γ^+^TNF-α^+^ CD8^+^ T cell population was the predominant polyfunctional T cell population after boost immunization.

### Antibody production after prime and boost administration

**Figure 8:**
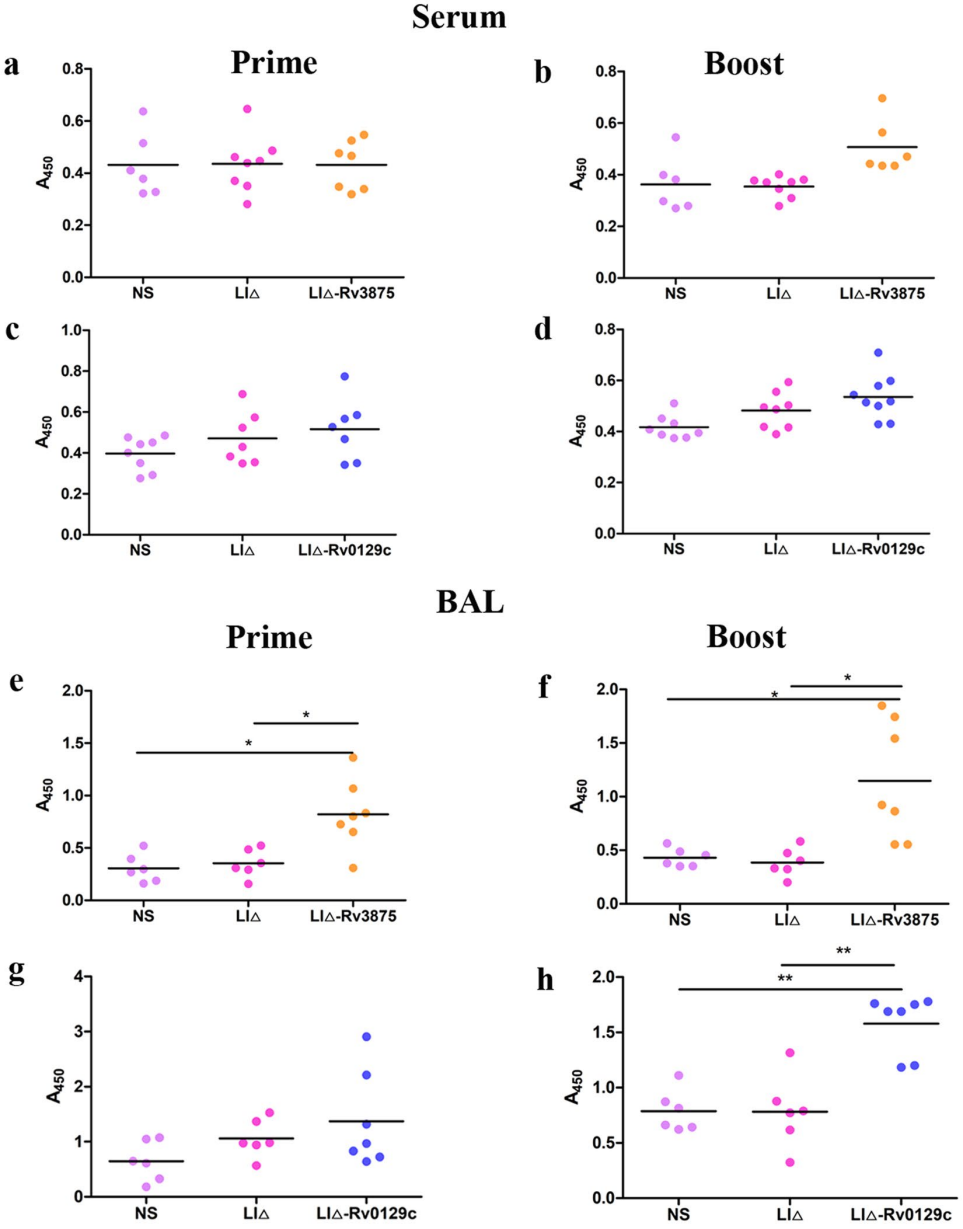
Intranasal administration of recombinant strains induced antigen-specific SIgA in BAL but did not induce antigen-specific IgG in serum. Two weeks after prime or boost administration, the levels of antigen-specific IgG in serum (**a–d**) and SIgA in BAL (**e**–**h**) were detected by ELISA. Serum samples from LI*Δ*-*Rv3875*-infected mice were diluted 1:400; serum samples from LI*Δ*-*Rv0129c*-infected mice were diluted 1:1000; BAL from both groups was diluted 1:1. Each point represents data for an individual mouse. *p < 0.05, **p < 0.01.

We then evaluated the capability of the two strains to induce antigen-specific antibodies in the pulmonary mucosal system and circulatory system (Fig. 8). Mice vaccinated with LI*Δ-Rv3875* had significantly higher SIgA levels in bronchoalveolar lavage (BAL) supernatant than those of the control groups after primary administration. After the boost administration, the SIgA level in the LI*Δ-Rv3875* immunization group was greatly increased. The SIgA level in the BAL supernatant of LI*Δ-Rv0129c-*immunized mice was not significantly higher than that in the controls after prime administration but sharply increased after boost administration. However, the two strains did not elicit notable production of immunogen-specific IgG in the serum. These results were consistent with the weak cellular immune responses in the spleen, which indicated that LI was restricted to the lung after intranasal administration.

## Discussion

In this study, we reported the antigen-specific lung-localized cellular immune response and pulmonary mucosal SIgA antibody secretion after intranasal administration of LI*Δ-Rv3875* or LI*Δ-Rv0129c*. It is not surprising that both strains failed to induce antigen-specific cellular immunity in the spleen because they did not establish a durable infection in the spleen. As we reported previously, when LI invaded the lung via the respiratory tract, the typical infection lesions that resulted seemed isolated and densely packed^18^. This phenomenon can be attributed to the unique feature of ivanolysin O (ILO). ILO expressed by LI has 76~78% homology to listeriolysin O (LLO)^19,20^, the major virulent protein of LM that functions in lysing the phagosome^21^. However, research has shown that ILO is not as strong as LLO at destroying the double vacuolar membrane, thus resulting in most of the LI being degraded in the phagosome because it is difficult to escape from phagosome to infect adjacent cells^22^. Therefore, LI in the lung is nearly impossible to disseminate to other organs. Furthermore, the LI strain used in this study was attenuated by knocking out the *actA* and *plcB* genes, making it more likely to be restricted to the lung tissue. When developing a mucosal vaccine, safety should be the primary concern. There was a report regarding the withdrawal of an inactivated influenza virosome vaccine (NasalFlu) containing an *Escherichia coli* heat labile toxin adjuvant (ELT) because it caused facial nerve paralysis^23^. This case, together with other similar cases, reminds us of the importance of safety for vaccines. Our results showed that LI*Δ-Rv3875* and LI*Δ-Rv0129c* were mostly restricted to the lung and were eliminated at 10 dpi. Additionally, the histological damage in the lungs caused by the strains was slight and recovered by 5 dpi.

The immune responses induced by intranasal administration of the two recombinant strains were quite different from those induced by intravenous administration. After intravenous administration, bacteria were delivered via circulation of the blood and directly entered peripheral immune organs such as the spleen and liver, where they were phagocytosed by phagocytes and dendritic cells, thus inducing efficient systemic immune responses^17^. Our earlier studies showed that wild-type LI mainly localized in the lung after intranasal administration^18^. Therefore, LI*Δ-Rv3875* and LI*Δ-Rv0129c* elicited lung-localized antigen-specific T cell immune responses and pulmonary mucosal SIgA. Such lung-localized immunity plays an important role in preventing respiratory diseases such as TB.

The prime-boost immune strategy is considered more effective than single-dose immunization. After the boost, a strong increase in the level of lung cytokines including IFN-γ, TNF-α and IL-17A, was observed. IFN-γ and TNF-α orchestrate initial monocyte and granulocyte recruitment and promote the antimicrobial activities of macrophages, which are considered to play critical roles in controlling Mtb infection^24^. IL-17A is mainly produced by Th17 cells that differentiate from CD4^+^ T cells. It was indicated that mucosal delivery of the tuberculosis vaccine can switch Th1 cells to Th17 cells^25^. Several studies show that IL-17A correlates with protection against TB at an early stage, given that IL-17A is capable of recruiting neutrophils and monocytes to the site of granuloma formation^26,27^. Intranasal vaccination with BCG triggers Mtb-specific mucosal immune response orchestrated by IL-17A, and thus confers better protection against Mtb challenge^5^. In addition, IL-17-producing CD4^+^ T cells in the lung are able to increase the production of chemokines to recruit more IFN-γ-producing CD4^+^ T cells and then restrict bacterial growth after Mtb challenge^26^. Taken together, IL-17 is helpful in controlling TB development. In the current study, the level of specific CD4^+^ IL-17A in lungs of mice that were primed by the strains was only significantly higher than in naïve mice (Fig. 5e), but it was strongly enhanced by a subsequent booster. Both strains induced a significantly higher percentage of CD4^+^ IL-17A than the vector control after boost vaccination (Fig. 6d), indicating that Mtb-specific CD4^+^ IL-17A was elicited. Polyfunctional Th1 cells have been suggested to amplify the protection against Mtb infection^28^. Moreover, polyfunctional CD8^+^ T cells have been proposed as a marker of protective immunity against Mtb^29^. Previous findings indicated that the polyfunctional T cells located in the lung were more effective than those in the spleen in providing protection against Mtb aerosol challenge^30^. Our data showed that both strains generated a high frequency of IFN-γ and TNF-α double-positive CD8 T cells in the lung (Fig. 7). Our results showed that Th1/Th17 and CD8^+^ T cellular immune responses in the lung were elicited by the recombinant LI strains.

Notably, our study suggested that intranasal administration of recombinant LI strains elicited promising pulmonary mucosal SIgA levels but poor IgG levels in the serum. The promising immune response in the pulmonary mucosa was consistent with the observation that the strains were mostly located in the lung after intranasal administration. Other researchers reported similar results. Qiu *et al*. reported that intranasal vaccination with rLMΔactA prfA* induced increased levels of immunogen-specific SIgA at mucosal interfaces but poor systemic IgG^31^. White *et al*. reported that the anti-vaccinia virus antibody response was not detected in the serum of animals after aerosol vaccination with MVA85A^32^. SIgA is the predominant Ig type in mucosal vaccine immunity. It was demonstrated that IgA-deficient mice were more susceptible to BCG infection^33^. Additionally, the pIgR−/− mice were more susceptible to mycobacterial infection compared with that of wild-type mice after Mtb infection^34^. These results reflect that SIgA plays an important role in protection against Mtb infection by blocking the entrance of bacilli into the lungs and modulating the pro-inflammatory response against Mtb infection^33^. The notable induction of pulmonary mucosal SIgA levels in this study suggests a promising immunogenicity of such LI-based Mtb delivery systems.

In conclusion, we suggest that LI might be a promising live vector for developing vaccines against respiratory pathogens via intranasal administration, given its low pathogenicity and the potency of vaccine-elicited immunity in the lung. Future studies should include a comprehensive investigation to determine whether immunity in the lung confers protection against infection. Our research provides a feasible method of establishing a mucosal vaccine against tuberculosis, which is helpful for expanding the application of LI as a vector in vaccine development.

## Materials and Methods

### Mice

Six-to eight-week-old female C57BL/6 J mice were purchased from the Animal Centre of Sichuan Province Hospital (Chengdu, China). All mice were maintained under specific pathogen-free conditions throughout the experiments at the Animal Centre of the School of Public Health at Sichuan University. Mouse experiments were performed according to the guidelines of the Animal Care and Use Committee of Sichuan University, and the protocols for mouse experiments were approved by this committee as well.

### Strains

LI*Δ*-*Rv3875* and LI*Δ*-*Rv0129c*, expressing Mtb ESAT-6 or Ag85C protein respectively were constructed as described in a previous study^17^. LI*Δ* was constructed and maintained in our laboratory as a vector control^17^. All strains were cultured at 37 °C with shaking at 180 r/m in brain heart infusion (BHI) broth.

### Western blot analysis

Proteins from LI*Δ-Rv3875* and LI*Δ-Rv0129c* were obtained by trichloroacetic acid precipitation as described previously^17^. Proteins were resolved by 10% sodium dodecyl sulfate polyacrylamide gel electrophoresis and transferred to polyvinylidene difluoride membranes. Following incubation with Tris-buffered saline containing 0.1% Tween-20 with 5% skim milk, the blots were probed with anti-ESAT6 (Abcam, USA) at a dilution of 1: 5 000 and anti-*Mycobacterium tuberculosis* Ag85 (Abcam, USA) at a dilution of 1: 500 overnight at 4 °C. Following horseradish peroxidase (HRP)-conjugated anti-mouse secondary antibody incubation (1:1000) (Beyotime Institute of Biotechnology, China), protein bands were visualized using Super Signal West Pico (Thermo Scientific, USA).

### Immunization and sampling

Four groups of randomly divided C57BL/6 J mice were intranasally inoculated with 10^8^ CFU of LI*Δ-Rv3875* (126 mice), LI*Δ-Rv0129c* (126 mice), or LI*Δ* (42 mice) in a volume of 10 μl or 10 μl of normal saline (48 mice). Boost immunization was performed four weeks later using the same dose of the same strains as the prime immunization (Fig. S1). We chose 10^8^ CFU as the immunization dose because a previous experiment showed that this dose was the maximum safe dose (Fig. S2). At 1, 2, 3, 5, 8 and 10 days post-prime immunization or post-boost immunization, six mice from the LI*Δ-Rv3875* or LI*Δ-Rv0129c* group were sacrificed to collect the liver, spleen and lung for the determination of the bacterial load in organs. Additionally, at 1 and 5 days post-prime immunization or post-boost immunization, six mice from the LI*Δ-Rv3875* and LI*Δ-Rv0129c* groups were sacrificed for histopathological analysis. Six mice from the naïve group were randomly chosen for histopathological analysis as a blank control. The histopathologic evaluator was blinded to the group information.

Two weeks after prime or boost immunization, the mice were sacrificed, and blood, lung, spleen and BAL were collected for subsequent examinations. Blood was collected through retro-orbital bleeding. After centrifugation, serum was isolated and stored at −80 °C for subsequent ELISA analysis of IgG. The lungs were removed and digested with 1 mg/ml collagenase I (Sigma, USA) and 0.1 mg/ml DNase I (Sigma, USA) to harvest lung cell suspensions for cytokine detection. Splenocyte suspensions for cytokine detection were prepared according to a previous study^17^. BAL was collected by making a small incision in the trachea and injecting 1 ml pre-cold phosphate buffer solution (PBS) with an indwelling needle. Then, the BAL supernatant was separated from cells by centrifugation and was frozen at −80 °C for detection of SIgA.

### Determination of bacterial load in organs

The liver, spleen and lung were harvested and homogenized in sterilized PBS containing 0.1% Triton X-100 as previously described^17^, diluted with PBS and plated on BHI agar. Colonies were counted after 24~48 h.

### Histopathologic analysis

The liver, spleen and lung were fixed in 10% buffered formalin, dehydrated and embedded in paraffin. Sections of 5 mm were cut, stained with haematoxylin-eosin and examined under 100 × magnification.

### Intracellular cytokine staining

A total of 2.5 × 10^6^ lung cells or splenocytes were added to 96-well microplates. Cells from LI*Δ-Rv3875-*infected mice and from LI*Δ-Rv0129c-*infected mice were stimulated with ESAT-6_1-20_ or Ag85C peptide complex (Ag85C _21–20_ and Ag85C_81–100_), respectively, at 37 °C and 5% CO_2_ for 4 h. Cells from the LI*Δ* and NS groups were stimulated with a mixture of all of the above mentioned peptides. For each well, the corresponding unstimulated cell well served as a blank control. Then, the cells were stained with FIFC-anti-CD3 and PerCP-anti-CD4 mAbs at 4 °C for 30 min. After permeabilization, the cells were stained with PE-anti-IFN-γ, APC-Cy7-anti-TNF-α and APC-anti-IL-17A mAbs and incubated at 4 °C for 45 min. After fixation, the cells were analysed by using a BD FACSVerse flow cytometer. A total of 5 × 10^5^ events were collected per well, and the results were calculated by using FlowJo software. For each well, the value was calculated by subtracting its corresponding blank control well.

### ELISA

Antigen-specific SIgA antibody in BAL supernatant was determined by ELISA. ELISA plates (Corning, USA) coated with 5 μg/ml ESAT-6 or 5 μg/ml Ag85C protein were incubated overnight at 4 °C and then blocked with 1% (w/v) bovine serum albumin in washing buffer for 2 h at 37 °C. BAL supernatant from LI*Δ-Rv3875-*infected mice diluted 1:1 was added into the ESAT-6-coated plates, and 1:1-diluted BAL supernatant from LI*Δ-Rv0129c-*infected mice was added into the Ag85C-coated plates. All samples were incubated for 1 h at 37 °C. Horseradish peroxidase-conjugated goat anti-mouse IgA (1:8000) was added and incubated for 1 h at 37 °C. The TMB substrate was added, and the reaction was stopped 20 min later. The optical density was measured at 450 nm.

Antigen-specific IgG antibody in serum was determined as described above. In brief, 1:400-diluted serum from LI*Δ-Rv3875-*infected mice was added into ESAT-6 coated plates, while 1:1000-diluted serum from LI*Δ-Rv0129c-*infected mice was added into Ag85C coated plates. Horseradish peroxidase-conjugated goat anti-mouse IgG (1:4000) was added. The optical density was measured at 450 nm.

### Statistical analysis

Statistical significance for comparisons of multiple groups was determined by one-way ANOVA using GraphPad Prism 5.0. Antigen-specific cytokines after prime and boost immunization for each group were compared by Student’s *t* test. In all experiments, *P* < 0.05 was considered as significant.

## References

[1] Rodrigues LC, Diwan VK, Wheeler JG (1993). Protective effect of BCG against tuberculous meningitis and miliary tuberculosis: a meta-analysis. Int. J. Epidemiol..

[2] Colditz GA (1994). Efficacy of BCG vaccine in the prevention of tuberculosis. Meta-analysis of the published literature. JAMA..

[3] Abebe F (2012). Is interferon-gamma the right marker for bacille Calmette-Guerin-induced immune protection? The missing link in our understanding of tuberculosis immunology. Clin. Exp. Immunol..

[4] Beverley PC, Sridhar S, Lalvani A, Tchilian EZ (2014). Harnessing local and systemic immunity for vaccines against tuberculosis. Mucosal Immunol..

[5] Aguilo N (2016). Pulmonary but not sbcutaneous delivery of BCG vaccine confers protection to tuberculosis-susceptible mice by an interleukin 17-dependent mechanism. J. Infect. Dis..

[6] Perdomo C (2016). Mucosal BCG vaccination induces protective lung-resident memory T cell populations against tuberculosis. MBio..

[7] Khan SH, Badovinac VP (2015). Listeria monocytogenes: a model pathogen to study antigen-specific memory CD8 T cell responses. Semin. Immunopathol..

[8] Zenewicz LA, Shen H (2007). Innate and adaptive immune responses to Listeria monocytogenes: a short overview. Microbes Infect..

[9] Stavru F, Archambaud C, Cossart P (2011). Cell biology and immunology of Listeria monocytogenes infections: novel insights. Immunol. Rev..

[10] Le DT, Dubenksy TW, Brockstedt DG (2012). Clinical development of Listeria monocytogenes-based immunotherapies. Semin. Oncol..

[11] Miller EA (2015). Attenuated Listeria monocytogenes vectors overcome suppressive plasma factors during HIV infection to stimulate myeloid dendritic cells to promote adaptive immunity and reactivation of latent virus. AIDS Res. Hum. Retroviruses..

[12] Deng W (2018). Recombinant Listeria promotes tumor rejection by CD8(+) T cell-dependent remodeling of the tumor microenvironment. Proc. Natl. Acad. Sci. USA.

[13] Wood LM, Paterson Y (2014). Attenuated Listeria monocytogenes: a powerful and versatile vector for the future of tumor immunotherapy. Front. Cell Infect. Microbiol..

[14] Roberts AJ, Wiedmann M (2003). Pathogen, host and environmental factors contributing to the pathogenesis of listeriosis. Cell Mol. Life Sci..

[15] Buchrieser. C (2011). Complete genome sequence of the animal pathogen Listeria ivanovii, which provides insights into host specificities and evolution of the genus Listeria. J. Bacteriol..

[16] Disson O, Lecuit M (2013). *In vitro* and *in vivo* models to study human listeriosis: mind the gap. Microbes Infect..

[17] Lin Q (2015). Construction of two Listeria ivanovii attenuated strains expressing Mycobacterium tuberculosis antigens for TB vaccine purposes. J. Biotechnol..

[18] Zhou M (2016). Listeria ivanovii infection in mice: restricted to the liver and lung with limited replication in the spleen. Front. Microbiol..

[19] Vazquez-Boland JA, Dominguez L, Rodriguez-Ferri EF, Suarez G (1989). Purification and characterization of two Listeria ivanovii cytolysins, a sphingomyelinase C and a thiol-activated toxin (ivanolysin O). Infect. Immun..

[20] Haas A, Dumbsky M, Kreft J (1992). Listeriolysin genes: complete sequence of ilo from Listeria ivanovii and of lso from Listeria seeligeri. Biochim. Biophys. Acta..

[21] Hamon MA, Ribet D, Stavru F, Cossart P (2012). Listeriolysin O: the Swiss army knife of Listeria. Trends Microbiol..

[22] Frehel C (2003). Capacity of ivanolysin O to replace listeriolysin O in phagosomal escape and *in vivo* survival of Listeria monocytogenes. Microbiology..

[23] Mutsch M (2004). Use of the inactivated intranasal influenza vaccine and the risk of Bell’s palsy in Switzerland. N. Engl. J. Med..

[24] Walzl G, Ronacher K, Hanekom W, Scriba TJ, Zumla A (2011). Immunological biomarkers of tuberculosis. Nat. Rev. Immunol..

[25] Orr MT (2015). Mucosal delivery switches the response to an adjuvanted tuberculosis vaccine from systemic TH1 to tissue-resident TH17 responses without impacting the protective efficacy. Vaccine..

[26] Khader SA (2007). IL-23 and IL-17 in the establishment of protective pulmonary CD4+ T cell responses after vaccination and during Mycobacterium tuberculosis challenge. Nat. Immunol..

[27] Okamoto Yoshida Y (2010). Essential role of IL-17A in the formation of a mycobacterial infection-induced granuloma in the lung. J. Immunol..

[28] Lewinsohn DA, Lewinsohn DM, Scriba TJ (2017). Polyfunctional CD4(+) T cells as targets for tuberculosis vaccination. Front. Immunol..

[29] Prezzemolo T (2014). Functional signatures of human CD4 and CD8 T cell responses to Mycobacterium tuberculosis. Front. Immunol..

[30] Forbes EK (2008). Multifunctional, high-level cytokine-producing Th1 cells in the lung, but not spleen, correlate with protection against Mycobacterium tuberculosis aerosol challenge in mice. J. Immunol..

[31] Qiu J (2011). Intranasal vaccination with the recombinant Listeria monocytogenes DeltaactA prfA* mutant elicits robust systemic and pulmonary cellular responses and secretory mucosal IgA. Clin. Vaccine. Immunol..

[32] White AD (2013). Evaluation of the safety and immunogenicity of a candidate tuberculosis vaccine, MVA85A, delivered by aerosol to the lungs of macaques. Clin. Vaccine. Immunol..

[33] Rodriguez A (2005). Role of IgA in the defense against respiratory infections IgA deficient mice exhibited increased susceptibility to intranasal infection with Mycobacterium bovis BCG. Vaccine..

[34] Tjarnlund A (2006). Polymeric IgR knockout mice are more susceptible to mycobacterial infections in the respiratory tract than wild-type mice. Int. Immunol..

